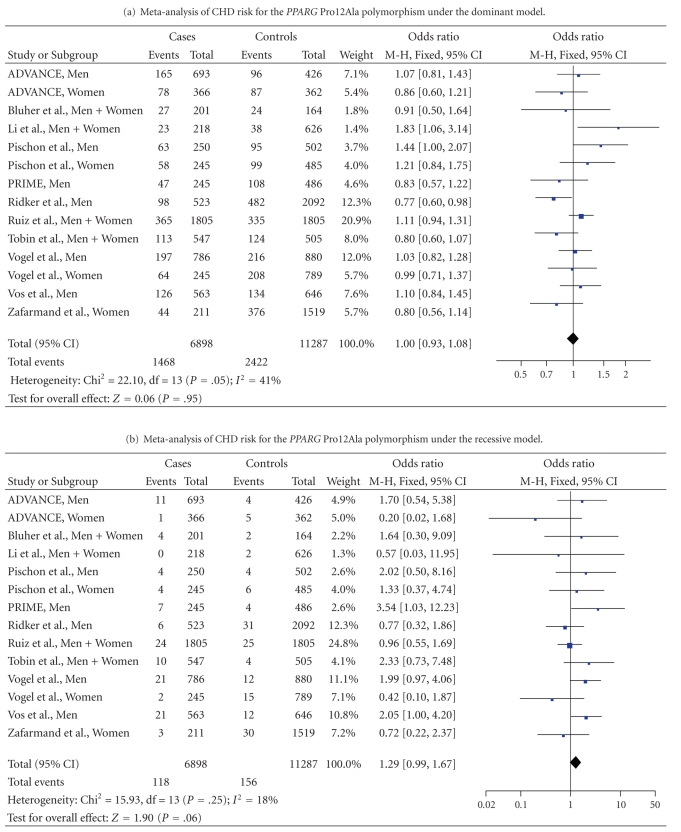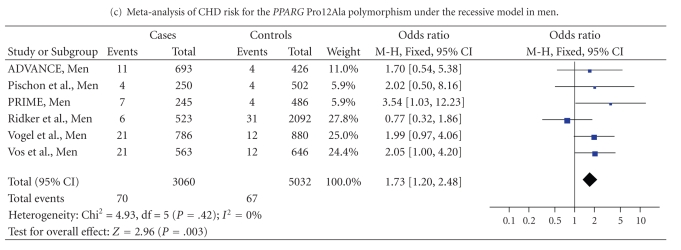# Peroxisome Proliferator-Activated Receptor Gamma Polymorphisms and Coronary Heart Disease

**DOI:** 10.1155/2009/543746

**Published:** 2009-12-01

**Authors:** Jean Dallongeville, Carlos Iribarren, Jean Ferrières, Liisa Lyon, Alun Evans, Alan S. Go, Dominique Arveiler, Stephen P. Fortmann, Pierre Ducimetière, Mark A. Hlatky, Philippe Amouyel, Audrey Southwick, Thomas Quertermous, Aline Meirhaeghe

**Affiliations:** ^1^Service d'Epidémiologie et de Santé Publique, Institut Pasteur de Lille, Lille; INSERM, U744, Lille; Université Nord de France, Lille; UDSL, 59019 Lille, France; ^2^Kaiser Permanente Division of Research, Oakland, CA 94612, USA; ^3^INSERM, U558, Toulouse, Department of Epidemiology, Paul Sabatier-Toulouse Purpan University, 31073 Toulouse, France; ^4^The Department of Epidemiology and Public Health, Queen's University Belfast, Belfast BT71NN, Northern Ireland, UK; ^5^Department of Epidemiology and Public Health, EA 3430, University of Strasbourg, Faculty of Medicine, 67085 Strasbourg, France; ^6^Stanford Prevention Research Center, Stanford University School of Medicine, Stanford, CA 94305-5705, USA; ^7^INSERM, U780, 94807 Villejuif, Hôpital Kremlin-Bicêtre, France; ^8^Department of Health Research and Policy, Stanford University School of Medicine, Stanford, CA 94305, USA; ^9^Stanford Human Genome Center, Stanford, CA 94304, USA; ^10^Falk Cardiovascular Research Center, Stanford Falk Cardiovascular Research Building, Stanford, CA 94305-5406, USA

## Abstract

Single nucleotide polymorphisms (SNPs) in the peroxisome proliferator-activated receptor *γ* (*PPARG*) gene have been associated with cardiovascular risk factors, particularly obesity and diabetes. We assessed the relationship between 4 *PPARG* SNPs (C-681G, C-689T, Pro12Ala, and C1431T) and coronary heart disease (CHD) in the PRIME (249 cases/494 controls, only men) and ADVANCE (1,076 cases/805 controls, men or women) studies. In PRIME, homozygote individuals for the minor allele of the *PPARG* C-689T, Pro12Ala, and C1431T SNPs tended to have a higher risk of CHD than homozygote individuals for the frequent allele (adjusted OR [95% CI] = 3.43 [0.96–12.27], *P* = .058, 3.41 [0.95–12.22], *P* = .060 and 5.10 [0.99–26.37], *P* = .050, resp.). No such association could be detected in ADVANCE. Haplotype distributions were similar in cases and control in both studies. A meta-analysis on the Pro12Ala SNP, based on our data and 11 other published association studies (6,898 CHD cases/11,287 controls), revealed that there was no evidence for a significant association under the dominant model (OR = 0.99 [0.92–1.07], *P* = .82). However, there was a borderline association under the recessive model (OR = 1.29 [0.99–1.67], *P* = .06) that became significant when considering men only (OR = 1.73 [1.20–2.48], *P* = .003). In conclusion, the *PPARG* Ala12Ala genotype might be associated with a higher CHD risk in men but further confirmation studies are needed.

## 1. Introduction

The peroxisome proliferator-activated receptor *γ* (PPARG) is a nuclear hormone receptor that dimerizes with the retinoid X receptor (RXR) to regulate target genes involved in adipocyte differentiation and insulin sensitization [1]. Activation of PPARG with thiazolidinediones is used to stimulate insulin sensitivity in the treatment of type 2 diabetes. PPARG also plays a role in macrophage, malignant breast epithelial, colon cancer cell differentiation, and lipid homeostasis [2] and has been shown to control the expression of proinflammatory genes in vascular cell models [3]. This suggests that PPARG may contribute to the pathogenesis of the atherosclerotic plaque physiology at a very early stage. 

Prior investigations have analyzed the relationship between common *PPARG* SNPs and various metabolic disorders. The Pro12Ala substitution in the specific exon B of PPARG2 isoform, which contributes to a lower PPARG2 activity in vitro, was associated with a decreased risk of type 2 diabetes [4]. Obese carriers of the minor allele of the *PPARG* C1431T polymorphism in exon 6 presented higher plasma leptin levels than noncarriers [5]. The C-681G and C-689T polymorphisms in the second and third *PPARG *promoter, respectively, were both associated with higher body weight and plasma LDL concentrations [6, 7]. Finally, haplotype analyses showed that a particular combination of these 4 polymorphisms was more frequent in patients with a metabolic syndrome than in control subjects [8]. Altogether, these observations suggest that genetic variability at the *PPARG* locus could affect cardiovascular risk.

Early studies analyzed the association between the *PPARG* Pro12Ala SNP and coronary heart disease (CHD) in cohorts from North America. These investigations yielded inconsistent results [9, 10]. Furthermore, a higher risk of coronary heart disease was reported in obese subjects carrying the Ala12 allele that needed confirmation [10]. Therefore, the goal of the present study was to explore the association between 4 *PPARG* SNPs, including the Pro12Ala SNP, and CHD risk in two independent studies among white subjects. We also performed haplotype analyses to evaluate whether a particular combination of alleles could better explain the effect of *PPARG* genetic variability on CHD risk and assessed whether overweight and obesity could modulate this risk. Finally, we performed a meta-analysis of published studies to date, focusing on the *PPARG* Pro12Ala polymorphism and CHD risk.

## 2. Methods

### 2.1. PRIME Study

The PRIME (Prospective Study of Myocardial Infarction) study is a prospective cohort study designed to identify risk factors for CHD [11]. Details on recruitment, baseline examination, and follow-up of the PRIME Study have been previously reported [12]. Overall, 9758 middle-aged men aged 50–59 years and free of CHD at baseline were recruited in Lille, Strasbourg, and Toulouse in France (*n* = 7399) and Belfast in Northern Ireland (*n* = 2359) between 1991 and 1993 and followed up for 5 years for the occurrence of first CHD events including coronary death, nonfatal myocardial infarction, and stable or unstable angina pectoris. 

#### 2.1.1. General Characteristics

Subjects who agreed to take part in the study were given a morning appointment and asked to fast for at least 12 hours. A full description of clinical and laboratory measurements has been published elsewhere [11, 12]. Briefly, a health questionnaire self-administered by subjects at their homes was subsequently checked by trained interviewers at the clinic. It covered a broad range of clinical information, including family and personal clinical histories, tobacco consumption, and drug intake. Blood pressure was measured twice in the sitting position with the same automatic device (Spengler SP9). A 12-lead ECG was also recorded. Plasma lipids analyses were centralized (SERLIA INSERM U325, Institut Pasteur de Lille, France). Overweight and obesity were defined as BMI ≥25 kg/m^2^ and BMI ≥30 kg/m^2^, respectively.

#### 2.1.2. Biochemical Measurements

A subset of biochemical measurements was performed on the entire cohort at baseline. Total cholesterol and triglycerides were measured by enzymatic methods using commercial kits in an automatic analyzer (Boehringer, Mannheim, Germany). High-density lipoprotein (HDL) cholesterol was determined after precipitation of Apo-lipoprotein B by an enzymatic method (Boehringer). Low-density lipoprotein (LDL) cholesterol was calculated according to the Friedewald formula. Insulin was assayed by competitive radioimmunoassay (Sanofi-Diagnostic Pasteur, France) in cases and matched controls using plasma samples collected at baseline and stored in liquid nitrogen.

During the follow-up, subjects were contacted annually by letter and asked to complete a clinical event questionnaire. For all subjects reporting a possible event, clinical information was sought directly from the hospital or general practitioner records. All details on ECGs, hospital admissions, enzymes, surgical intervention, angioplasty, treatments, and so forth were collected. Death certificates were checked for supporting clinical and postmortem information on cause of death. Whenever possible, circumstances of death were obtained from the practitioner or the family. A Medical Committee comprising one member from each PRIME Center and the Coordinating Center, and three cardiologists (two from France and one from the UK), was established to provide an independent validation of coronary events. A description of the coronary end point definitions has been published recently [12]. The five-year follow-up was completed in 98,6% of the French participants and 99,2% of the Northern-Irish participants. 

A nested case-control study within the PRIME prospective cohort study was mounted using the baseline plasma samples of 335 study participants who subsequently developed a future coronary ischemic event during follow-up and of 670 matched controls (2 controls per case). Matched controls were study participants recruited in the same center, on the same day (±3 days), of the same age (±3 years) as the case and free of CHD at the time of the ischemic event of the case. Subjects with incomplete data were excluded. If a case was excluded, his two matched controls were also excluded, and if the two matched controls for a case were excluded, the corresponding case was also excluded. A total of 249 cases and 494 controls were used in the present study.

### 2.2. The ADVANCE Study

The Atherosclerotic Disease, VAscular FuNction, and genetiC Epidemiology (ADVANCE) study recruited, between October 2001 and December 2003, a total of 3,179 subjects from multiple race/ethnic background into 5 cohorts: a cohort of subjects with clinically significant CAD at a young age (≤45 years for males, ≤55 years for females), a cohort of subjects with incident stable angina at an older age, a cohort of subjects with incident acute myocardial infarction (AMI) at an older age, a cohort of young subjects with no history of CAD, and a cohort of subjects aged 60 to 72 with no history of CAD, ischemic stroke, or peripheral arterial disease (PAD). Eligible subjects were identified using the Kaiser Permanente of Northern California (KPNC) electronic databases and those who agreed to participate were interviewed and examined at one of several clinics in the San Francisco Bay Area. A sixth cohort of young subjects with no history of CAD included 479 participants in the Coronary Artery Risk Development in Young Adults (CARDIA) Study [13] originally recruited through KPNC and attending the study's year 15 examination cycle in 2000-2001. A detailed description of the source population for all cohorts has been published elsewhere [14, 15]. The design of the ADVANCE study allowed for several case control comparisons. In this study, we included all white subjects with symptomatic early onset CAD (young cases), older subjects with either AMI or angina as first presentation of CHD (older cases), young controls, and older controls. This resulted in 1 076 CHD cases (706 in men, 370 in women) and 805 controls (433 male, 372 female). 

The ADVANCE study was approved by the Institutional Review Board at both Stanford University and Kaiser Permanente of Northern California (KPNC). Details of the methodology for risk factor assessment can be found elsewhere [14, 15].

### 2.3. Genotyping

The genotyping method in PRIME has been described previously [8]. The genotyping success rate was above 94% for each SNP. Genotyping in ADVANCE was performed at the Stanford Human Genome Center using the ABI 7900 TaqMan platform [16, 17]. The “no call rate” for genotypes was only 0.35% and the reproducibility was 99.9% in random samples with repeat genotypes.

### 2.4. Statistical Analyses

For ADVANCE, we used unconditional logistic regression because cases and controls were not individually matched. For PRIME, we used conditional logistic regression analysis to compare the distribution of genotypes between cases and controls and to estimate the odds ratio (OR) of CHD. The analyses were adjusted for age, educational level, alcohol consumption, physical activity, smoking status, history of diabetes, history of hypertension, and history of dyslipidemia. Additional analyses were performed to test the interaction between overweight or obesity and genotype by adding a cross-product term to the logistic regression model. We used general regression models and chi-square test to compare the clinical and biological characteristics of the subjects according to genotype in control subjects. Triglyceride values were log transformed for analyses. All analyses were performed using SAS software 8.2 version (SAS Institute, Cary, USA).

#### 2.4.1. Haplotype Analyses

Presence of linkage disequilibrium between the loci was tested using a log-likelihood-ratio test [18] and the degree of disequilibrium was expressed in terms of normalized difference *D*′ = *D*/*D*_max_ or *D*/*D*_min_ [19]. Haplotype frequencies were estimated using a stochastic version of the expectation-maximization algorithm as implemented in Thesias software [20, 21]. Differences in haplotype frequencies between cases and their respective controls were examined using a log-likelihood ratio statistic test which was computed from the estimated haplotype frequency log-likelihoods for the case and control groups separately.

#### 2.4.2. Meta-Analysis

In addition to the analysis of PRIME and ADVANCE, we identified all published prospective studies that assessed the relationship between the *PPARG* Pro12Ala polymorphism and CHD and conducted a meta-analysis of the findings. Inclusion criteria for events were a coronary event. Inclusion criteria for exposure variable were the *PPARG* Pro12Ala polymorphism. Searches were conducted in electronic databases (MEDLINE and EMBASE) from 1970 to June 2009. References from the extracted papers, reviews, and previous meta-analysis were also consulted to complete the data bank. The electronic search include both truncated free-text and MeSH. Used terms were “PPARG” or “peroxisome proliferator-activated receptor gamma” and “polymorphism” and “cardiovascular disease” or “CAD” or “CHD” or “coronary” or “MI” or “myocardial” or “ischemic heart disease.” No attempt was made to contact authors of nonpublished work or to find paper in other languages than English. When the data were not available in the appropriate format, the corresponding author of the paper was contacted to obtain the data. However, two studies were excluded because the data were not available in the appropriate format, that is, the number of subjects for the three genotype groups in cases and controls [22, 23]. Finally, 11 studies were selected for the meta-analysis. Therefore, the final data set, including our 3 samples, consisted of 14 independent studies comprising 6898 cases and 11 287 controls (Table 4).

For this meta-analysis, we combined data of all studies using Review Manager software release 5.0 (http://www.cc-ims.net/revman/) [24]. We estimated the overall effect by a Mantel–Haentzel fixed odds ratio.

## 3. Results

As expected, major cardiovascular risk factors such as smoking, history of hypertension, history of dyslipidemia, history of diabetes, and systolic and diastolic blood pressure levels differed between cases and controls in both PRIME and ADVANCE studies (Table 1).

The genotype distributions of the 4 *PPARG* SNPs respected the Hardy-Weinberg equilibrium in control subjects. In the control group, the frequencies of the *PPARG* -681G, -689T, Ala12, and 1431T alleles were similar in PRIME and ADVANCE. There were marginal differences in the genotype distribution of the C-689T (*P* = .04), Pro12Ala (*P* = .03), and C1431T (*P* = .04) SNPs between cases and controls in PRIME (Table 2(a)), mainly due to a higher frequency of homozygote subjects for the minor allele in cases compared with controls. 

The odds ratios (ORs) [95% CI] of CHD were calculated for heterozygotes, for homozygotes, and for carriers of the minor allele (dominant model), using both a crude model and an adjusted model (Table 2(a)). In PRIME, homozygote individuals for the minor allele of the *PPARG* C-689T, Pro12Ala, and C1431T SNPs tended to have a higher risk of CHD than homozygote individuals for the frequent allele (OR [95% CI] = 3.34 [0.98–11.45], *P* = .054, OR = 3.32 [0.97–11.39], *P* = .056 and OR = 5.93 [1.19–29.45], *P* = .029, resp.). After adjustment for age, educational level, smoking status, physical activity, alcohol intake, history of diabetes, history of hypertension, and history of dyslipidemia, the ORs were only slightly altered (OR = 3.43 [0.96–12.27], *P* = .058, 3.41 [0.95–12.22], *P* = .060 and 5.10 [0.99–26.37], *P* = .050 for the C-689T, Pro12Ala and C1431T SNPs, resp.). There was no significant association in ADVANCE, neither in men nor in women (Table 2(b) and (c)). Further analyses stratifying on overweight (<25 kg/m^2^ versus ≥25 kg/m^2^) or obesity (<30 kg/m^2^ versus ≥30 kg/m^2^) or adding a cross-product term into the model yielded no significant interaction (all *P* values for interaction term >0.1) in both studies (data not shown). 

In control subjects from PRIME and ADVANCE, there were very few significant differences in baseline characteristics between carriers and noncarriers of the *PPARG*-681G, -689T, Ala12, and 1431T alleles (see Tables 1, 2, 3, 4, and 5 in Supplementary Material available online at doi:10.155/2009/543746). Systolic blood pressure was significantly lower (*P* < .05) in carriers of the* PPARG*-681G, -689T and Ala12 alleles in PRIME (Supplementary Table 1). There was no statistically significant difference in mean BMI, LDL-cholesterol, or HDL-cholesterol levels between carriers and noncarriers of the minor allele of any SNP investigated. There was also no evidence for any statistically significant difference in the prevalence of reported hypertension, dyslipidemia, or diabetes between carriers and noncarriers of the minor allele of these SNPs.

The Pro12Ala and the C-681G, C-689T, and C1431T SNPs were in partial positive linkage disequilibrium (*D*′*  *range = +0.6 − +1.0, *r* ^2^ **range = 0.18–0.98). The C-689T and Pro12Ala SNPs were in perfect linkage disequilibrium (*D*′ = +1.0, *r* ^2^ = 0.98). The distribution of the 4 SNP -containing haplotypes was estimated in cases and controls (Table 3). Six haplotypes covered 98% of the possible haplotypes. The frequency of the haplotype composed of the 4 most frequent alleles was >70% in both cases and controls. There was no evidence for any statistically significant difference in haplotype distribution between cases and controls, neither in PRIME nor in ADVANCE (*P* > .38). Haplotype analyses were also conducted after stratification on overweight or obesity. In these analyses, there was no evidence of significant difference of haplotype distributions between cases and controls in overweight or lean, obese, or nonobese subjects (data not shown).

Pooled estimate of CHD odds ratios were calculated from published studies for the *PPARG* Ala12Pro SNP including 6898 cases and 11 287 controls. Funnel plot analysis revealed no evidence of publication bias. There was no significant association with CHD under the dominant genetic model (OR = 1.00 [0.93–1.08], *P* = .95) (Table 4(a)). There was significant heterogeneity in the meta-analysis (Chi² = 22.10, df = 13, *P* = .05) due to the study of Li et al., which was the only study to show a significant association between the Ala12 allele and a higher CHD risk. When removing this study from the meta-analysis (heterogeneity Chi² = 17.37, df = 12, *P* = .14), the odds ratio of CHD was 0.99 [0.92–1.07], *P* = .82. The ORs of CHD were similar in men (OR = 0.99, *P* = .91) and women (OR = 0.94, *P* = .52).

Using the recessive genetic model, there was a borderline association between the Ala12Ala genotype and CHD risk (OR = 1.29 [0.99–1.67], *P* = .06) (Table 4(b)). When stratifying on gender, Ala12Ala men had a significant higher risk of CHD (OR = 1.73 [1.20–2.48], *P* = .003) than men carrying the Pro12 allele (Table 4(c)). No such association could be detected in women (OR = 0.62 [0.31–1.24], *P* = .17) (data not shown).

## 4. Discussion

In the present study, we did not find any consistent association between the *PPARG* C-681G, C-689T, Pro12Ala, or C1431T SNPs or related haplotypes and CHD risk, neither in men nor in women, in the PRIME and ADVANCE studies. Furthermore, analyses stratified on overweight or obesity revealed no evidence that these *PPARG* variants influence CHD risk in overweight or obese men. These data do not support the hypothesis that *PPARG* C-681G, C-689T, Pro12Ala, and C1431T polymorphisms are major risk factors for CHD but does not rule out the possibility of an association of small magnitude, especially in homozygote Ala12Ala men.

PPARG is a ligand-activated transcription factor playing an important role in adipocyte differentiation, glucose homeostasis, and several vascular processes. In vitro studies have shown that the *PPARG* Ala12 allele decreases PPARG activation of reporter genes (4) and the -681G and -689T alleles are associated with lower PPARG promoter activity [4, 6, 7] suggesting that carriers of these alleles might have a lower PPARG-mediated activation of target genes than noncarriers. 

Earlier clinical and epidemiological studies have assessed the relationship between variability at the *PPARG* gene loci and CHD risk. The results of these investigations have been inconsistent with some studies showing a reduced risk of coronary heart disease [9, 22], other studies an increased risk [25–29] and some others reporting no association [10, 23, 30–33]. In contrast, three studies showed less intima-media carotid thickening in carriers of the *PPARG* Ala12 allele suggesting a possible relation between this variant and sub-clinical atherosclerosis [34–36]. 

We found no consistent association for none of the 4 *PPARG* SNPs (C-681G, C-689T, Pro12Ala, and C1431T) in PRIME and ADVANCE. Only in PRIME, we observed that men homozygotes for the minor allele of the C-689T, Pro12Ala, and C1431T SNPs had a higher risk of CHD than homozygotes for the frequent allele. This result was further confirmed in the meta-analysis including 3060 cases and 5032 controls that showed a higher risk of CHD in Ala12Ala men. Recent genome whole association studies failed to identify associations with *PPARG *SNPs [37–42]. Firstly, this might be explained by the gender-specific effect and the restrictive recessive genetic model of the *PPARG* association. Secondly, the 4 SNPs that were investigated in the present study may not be the culprit ones, but rather be in linkage disequilibrium with a causal mutation, resulting in a dilution of a possible association. 

*PPARG* SNPs may affect CHD risk through their association with cardiovascular risk factors. In the present study, we found no evidence for significant differences in major cardiovascular risk factors for example, body mass index, lipid levels, or history of diabetes, between carriers and noncarriers of the minor alleles. The only exception was a borderline association with systolic blood pressure level which was lower in *PPARG*-681G, -689T, and Ala12 carriers than in noncarriers in the PRIME study and could confer some protection against CHD. These findings are in agreement with other studies which showed lower levels of blood pressure or risk of hypertension in carriers of the minor allele of the *PPARG* Pro12Ala SNP [43–45]. However, these associations are inconsistent across studies as some also reported higher levels [46, 47] or not significantly different levels [48, 49] of blood pressure between carriers and noncarriers of the *PPARG* Ala12 allele. In this sample of apparently healthy men, the prevalence of hypertension was low (~18%) and the impact of the *PPARG*-681G, -689T, and Ala12 alleles on blood pressure was clinically limited.

An earlier study has reported a significant higher risk of MI and fatal CHD in overweight but not in normal weight subjects [10] suggesting that the impact of *PPARG* genotype may differ according to body fat mass. In the present study, which has a similar number of cases and controls, we found no evidence for such a relationship, suggesting that obesity is not a determinant of the association between *PPARG* genetic variability and CHD.

## 5. Conclusion

In the present study, there was no major consistent association between the *PPARG* C-681G, C-689T, Pro12Ala, and C1431T genotypes or related haplotypes and CHD risk. When combining our data with previous cohort studies, focusing on the *PPARG *Pro12Ala SNP, we found a significant association between the homozygote Ala12Ala genotype and a higher risk of CHD in men. Further studies are needed to definitely conclude.

##  The PRIME Study Group

The Strasbourg MONICA Project, Department of Epidemiology and Public Health, Faculty of Medicine, Strasbourg, France (D. Arveiler, B. Haas).

The Toulouse MONICA Project, INSERM U558, Department of Epidemiology, Paul Sabatier-Toulouse Purpan University, Toulouse, France (J. Ferrières, JB. Ruidavets).

The Lille MONICA Project, INSERM U744, Pasteur Institute, Lille, France (P. Amouyel, M. Montaye).

The Department of Epidemiology and Public Health, Queen's University, Belfast, Northern Ireland (A. Evans, J. Yarnell, F. Kee).

The Department of Atherosclerosis, INSERM U545, Lille, France (G. Luc, JM. Bard).

The Laboratory of Haematology, INSERM U626, La Timone Hospital, Marseilles, France (I. Juhan-Vague, Pierre Morange).

The Laboratory of Endocrinology, INSERM U563, Toulouse, France (B. Perret).

The Vitamin Research Unit, The University of Bern, Bern, Switzerland (F. Gey).

The Trace Element Laboratory, Department of Medicine, Queen's University Belfast, Northern Ireland (Jayne Woodside, Ian Young).

The DNA Bank, INSERM U525, Paris, France (F. Cambien).

The Coordinating Center, INSERM U780 (ex-U258), Paris-Villejuif, France (P. Ducimetière, A. Bingham).

## Supplementary Material

Supplementary Table 1: Baseline characteristics of controls according to *PPARG* genotypes in the PRIME Study.
Supplementary Table 2: Baseline characteristics of white controls according to the *PPARG* C-689T genotype in the ADVANCE Study.
Supplementary Table 3: Baseline characteristics of white controls according to the *PPARG* C-681G genotype in the ADVANCE Study.
Supplementary Table 4: Baseline characteristics of white controls according to the *PPARG* Pro12Ala genotype in the ADVANCE Study.
Supplementary Table 5: Baseline characteristics of white controls according to the *PPARG* C1431T genotype in the ADVANCE Study.

## Figures and Tables

**Table 1:** Baseline characteristics of subjects with incident CHD (case) and CHD-free (control) subjects in PRIME and ADVANCE.

	PRIME Men			ADVANCE Men			ADVANCE Women		
	Controls	Cases	P*	Controls	Cases	P*	Controls	Cases	P*
N	494	249		433	706		372	370	
Age (y)	55.1 ± 2.8	55.3 ± 3.0	.46	65.8 ± 3.3	61.5 ± 7.9	<.0001	61.5 ± 6.9	60.0 ± 8.8	.11
BMI (kg/m²)	26.7 ± 3.5	27.1 ± 3.4	.11	28.3 ± 4.4	29.1 ± 4.8	.005	27.4 ± 6.3	29.6 ± 7.2	<.0001
Waist girth (cm)	93.6 ± 10.1	95.0 ± 10.2	.067	99.4 ± 12.6	100.0 ± 12.0	.46	83.8 ± 13.6	90.4 ± 15.9	<.0001
Years at school (y)	11.26 ± 5	10.9 ± 3	.36	NA	NA		NA	NA	
Physically active (%)	19.6	19.6	.99	62.6	53.8	.004	63.2	45.7	<.0001
Current smokers (%)	30.6	19.7	.0019	6.8	8.4	.36	8.4	13.0	.04
Alcohol consumption (g/week)	236 ± 310	234 ± 323	.88	50 ± 140	20 ± 70	<.0001	20 ± 70	0 ± 30	<.0001
History of hypertension (%)	17.6	30.1	.0001	51.5	78.8	<.0001	41.7	79.2	<.0001
History of dyslipidemia (%)	28.5	34.9	.06	25.4	28.6	.46	11.6	26.2	<.0001
History of diabetes (%)	4.9	8.8	.036	15.5	23.0	<.0001	5.1	21.4	<.0001
Systolic BP (mm Hg)	13 ± 19	141 ± 2	<.0001	131 ± 16	120 ± 17	<.0001	124 ± 19	121 ± 20	.08
Diastolic BP (mm Hg)	84 ± 13	87 ± 12	.0008	75 ± 8	71 ± 9	<.0001	71 ± 9	69 ± 9	.0004
Total cholesterol (mg/dL)	225 ± 37	234 ± 39	.0008	202 ± 35	NA	—	210 ± 36	NA	—
LDL-cholesterol (mg/dL)	146 ± 34	157 ± 33	.0001	124 ± 30	NA	—	124 ± 30	NA	—
HDL-cholesterol (mg/dL)	47 ± 12	43 ± 11	<.0001	49 ± 14	NA	—	62 ± 17	NA	—

Data are expressed as means ± SD or percentages. *T-test for continuous variables and Chi-square test for categorical variables. NA: Not available. BP: blood pressure.

**(a):** 

PRIME Men							
				Model 1		Model 2	
	Controls	Cases	P*	OR [95% CI]	*P*	OR [95% CI]	*P*
C-681G (n)	484	243					
CC, n (%)	286 (59.1)	146 (60.1)		reference		reference	
CG, n (%)	174 (35.9)	79 (32.5)	.32	0.88 [0.64–1.22]	.44	0.90 [0.64–1.26]	.53
GG, n (%)	24 (5.0)	18 (7.4)		1.54 [0.79–3.00]	.20	1.64 [0.82–3.30]	.16
CG+GG, n (%)	198 (40.9)	97 (39.9)		0.96 [0.71–1.31]	.82	0.97 [0.71–1.34]	.88
C-689T (n)	484	242					
CC, n (%)	374 (77.3)	193 (79.7)		reference		reference	
CT, n (%)	106 (21.9)	42 (17.4)	.04	0.76 [0.51–1.13]	.18	0.74 [0.49–1.13]	.16
TT, n (%)	4 (0.8)	7 (2.9)		3.34 [0.98–11.45]	.054	3.43 [0.96–12.27]	.058
CT+TT, n (%)	110 (22.7)	49 (20.3)		0.89 [0.61–1.30]	.54	0.87 [0.58–1.29]	.48
Pro12Ala (n)	486	245					
CC, n (%)	378 (77.8)	198 (80.8)		reference		reference	
CG, n (%)	104 (21.4)	40 (16.3)	.03	0.74 [0.49–1.10]	.14	0.72 [0.47–1.10]	.12
GG, n (%)	4 (0.8)	7 (2.9)		3.32 [0.97–11.39]	.056	3.41 [0.95–12.22]	.060
CG+GG, n (%)	108 (22.2)	47 (19.2)		0.85 [0.58–1.25]	.40	0.83 [0.55–1.24]	.36
C1431T (n)	482	241					
CC, n (%)	383 (79.5)	189 (78.4)		reference		reference	
CT, n (%)	97 (20.1)	46 (19.1)	.04	0.94 [0.64–1.38]	.76	0.89 [0.60–1.33]	.58
TT, n (%)	2 (0.4)	6 (2.5)		5.93 [1.19–29.45]	.029	5.10 [0.99–26.37]	.050
CT+TT, n (%)	99 (20.5)	52 (21.6)		1.01 [0.69–1.46]	.98	0.95 [0.64–1.40]	.78

**(b):** 

ADVANCE Men							
				Model 1		Model 2	
	Controls	Cases	P*	OR [95% CI]	*P*	OR [95% CI]	*P*
C-681G (n)	420	694					
CC, n (%)	238 (56.7)	395 (56.9)		reference		reference	
CG, n (%)	159 (37.9)	264 (38.1)	.95	1.00 [0.78–1.29]	.99	1.03 [0.77–1.37]	.85
GG, n (%)	23 (5.4)	35 (5.0)		0.92 [0.53–1.59]	.76	0.85 [0.46–1.60]	.62
CG+GG, n (%)	182 (43.3)	299 (43.1)		0.99 [0.77–1.26]	.93	1.00 [0.76–1.32]	.99
C-689T (n)	423	687					
CC, n (%)	326 (77.1)	522 (76.0)		reference		reference	
CT, n (%)	93 (22.0)	154 (22.4)	.68	1.03 [0.77–1.39]	.82	1.00 [0.72–1.38]	.98
TT, n (%)	4 (0.9)	11 (1.6)		1.72 [0.54–5.44]	.35	1.87 [0.48–7.33]	.37
CT+TT, n (%)	97 (22.9)	165 (24.0)		1.06 [0.80–1.41]	.68	0.99 [0.78–1.26]	.94
Pro12Ala (n)	426	693					
CC, n (%)	330 (77.5)	528 (76.2)		reference		reference	
CG, n (%)	92 (21.6)	154 (22.2)	.62	1.05 [0.78–1.40]	.76	1.03 [0.74–1.42]	.88
GG, n (%)	4 (0.9)	11 (1.6)		1.72 [0.54–5.44]	.36	1.90 [0.49–7.41]	.36
CG+GG, n (%)	96 (22.5)	165 (23.8)		1.07 [0.81–1.43]	.62	1.06 [0.77–1.45]	.74
C1431T (n)	426	687					
CC, n (%)	325 (76.3)	530 (77.2)		reference		reference	
CT, n (%)	98 (23.0)	147 (21.4)	.50	0.92 [0.69–1.23]	.57	0.87 [0.63–1.21]	.41
TT, n (%)	3 (0.7)	10 (1.4)		2.01 [0.55–7.36]	.29	1.76 [0.43–7.16]	.43
CT+TT, n (%)	101 (23.7)	157 (22.8)		0.95 [0.71–1.25]	.73	0.90 [0.65–1.24]	.52

**(c):** 

ADVANCE Women							
				Model 1		Model 2	
	Controls	Cases	P*	OR [95% CI]	*P*	OR [95% CI]	*P*
C-681G (n)	359	365					
CC, n (%)	189 (52.7)	203 (55.6)		reference		reference	
CG, n (%)	148 (41.2)	144 (39.5)	.64	0.91 [0.67–1.23]	.52	0.97 [0.68–1.38]	.86
GG, n (%)	22 (6.1)	18 (4.9)		0.76 [0.40–1.47]	.42	0.64 [0.30–1.4]	.26
CG+GG, n (%)	170 (47.3)	162 (44.4)		0.89 [0.66–1.19]	.42	0.92 [0.65–1.29]	.62
C-689T (n)	360	365					
CC, n (%)	274 (76.1)	285 (78.1)		reference		reference	
CT, n (%)	81 (22.5)	79 (21.6)	.26	0.94 [0.66–1.33]	.72	1.07 [0.71–1.6]	.74
TT, n (%)	5 (1.4)	1 (0.3)		0.19 [0.02–1.66]	.13	0.17 [0.02–1.54]	.12
CT+TT, n (%)	86 (23.9)	80 (21.9)		0.89 [0.63–1.26]	.53	1.00 [0.67–1.49]	.99
Pro12Ala (n)	362	366					
CC, n (%)	275 (76.0)	288 (78.7)		reference		reference	
CG, n (%)	82 (22.6)	77 (21.0)	.22	0.90 [0.63–1.28]	.54	1.02 [0.68–1.53]	.93
GG, n (%)	5 (1.4)	1 (0.3)		0.19 [0.02–1.65]	.13	0.168 [0.02–1.51]	.11
CG+GG, n (%)	87 (24.0)	78 (21.3)		0.86 [0.60–1.21]	.38	0.99 [0.67–1.49]	.80
C1431T (n)	366	367					
CC, n (%)	278 (76.0)	281 (76.6)		reference		reference	
CT, n (%)	81 (22.1)	83 (22.6)	.50	1.01 [0.72–1.44]	.94	1.08 [0.72–1.63]	.70
TT, n (%)	7 (1.9)	3 (0.8)		0.42 [0.11–1.66]	.21	0.30 [0.07–1.32]	.11
CT+TT, n (%)	88 (24.0)	86 (23.4)		0.97 [0.69–1.36]	.85	1.00 [0.67–1.49]	.96

*P value for a global test of significance. Model 1: crude OR. Model 2: OR adjusted for age, BMI, educational level, smoking status, physical activity, alcohol intake, history of diabetes, history of hypertension, and history of dyslipidemia.

**Table 3:** Estimate of *PPARG* haplotype frequencies in cases and controls.

C-681G	C-689T	Pro12Ala	C1431T	Controls	Cases	*P*
PRIME Men						
1	1	1	1	0.72	0.72	
2	1	1	1	0.11	0.11	
2	2	2	2	0.08	0.09	.53
2	2	2	1	0.04	0.03	
1	1	1	2	0.03	0.03	
2	1	1	2	0.01	0.02	

ADVANCE Men						
1	1	1	1	0.74	0.73	
2	1	1	1	0.11	0.10	
2	2	2	2	0.09	0.08	.38
2	2	2	1	0.03	0.04	
1	1	1	2	0.02	0.02	
2	1	1	2	0.02	0.01	

ADVANCE Women						
1	1	1	1	0.71	0.72	
2	1	1	1	0.13	0.13	
2	2	2	2	0.08	0.08	.70
2	2	2	1	0.04	0.03	
1	1	1	2	0.03	0.03	
2	1	1	2	0.01	0.01	

Only haplotypes with a frequency >1% are displayed. 1 and 2 represent the frequent and minor alleles, respectively. *P* value is for the global effect (5 df).

**Table 4:**