# Supplementary material for: Peroxisome Proliferator-Activated Receptor Gamma Polymorphisms and Coronary Heart Disease
Source: PPAR Res. 2009 Dec 1;2009:543746. doi: 10.1155/2009/543746 (PMC2792957; doi:10.1155/2009/543746)
Supplement: Supplementary file 1 — Supplementary Table 1: Baseline characteristics of controls according to PPARG genotypes in the PRIME Study. Supplementary Table 2: Baseline characteristics of white controls according to the PPARG C-689T genotype in the ADVANCE Study. Supplementary Table 3: Baseline characteristics of white controls according to the PPARG C-681G genotype in the ADVANCE Study. Supplementary Table 4: Baseline characteristics of white controls according to the PPARG Pro12Ala genotype in the ADVANCE Study. Supplementary Table 5: Baseline characteristics of white controls according to the PPARG C1431T genotype in the ADVANCE Study. [file 543746.f1.pdf]

Suppl Table 1. Baseline characteristics of controls according to *PPARG* genotypes in the PRIME Study

	C-681G			C-689T			Pro12Ala			
	CC	G+	*p**	CC	T+	*p**	Pro12Pro	Ala+	*p**	CC
N	286	198		374	110		378	108		383
Age (y)	55.2±2.8	55.0±2.8	*0.38*	55.0±2.8	55.2±3.0	*0.12*	55.0±2.8	55.2±3.0	*0.11*	55.1±2.7
BMI (kg/m²)	26.7±3.6	26.6±3.4	*0.56*	26.6±3.4	26.8±4.0	*0.80*	26.6±3.4	26.7±4.1	*0.71*	26.5±3.2
Waist girth (cm)	93.8±10.4	93.4±9.7	*0.66*	93.5±9.8	94.0±11.4	*0.67*	93.5±9.8	93.9±11.4	*0.74*	93.2±9.3
Years at school (y)	11.4±6.0	11.0±3.0	*0.73*	11.3±5.5	10.9±2.5	*0.44*	11.3±5.5	11.0±2.5	*0.71*	11.1±3.2
Physicaly active (%)	18.5	20.2	*0.46*	19.5	19.1	*0.37*	19.6	19.4	*0.37*	19
Current smokers (%)	29.4	32.3	*0.14*	30.2	31.8	*0.34*	30.9	30.5	*0.63*	32.1
Alcohol consumption (g/week)	232±299	243±330	*0.41*	225±285	266±386	*0.09*	227±285	266±389	*0.09*	230±304
History of hypertension (%)	20.6	13.6	*0.37*	18.4	15.4	*0.99*	18.5	14.8	*0.83*	18.5
History of dyslipidemia (%)	27.3	29.8	*0.10*	28.6	28.2	*0.72*	28.6	27.8	*0.72*	27.7
History of diabetes (%)	4.5	5.0	*0.18*	5.1	4.5	*0.21*	5.0	4.6	*0.21*	4.7
Total cholesterol (mg/dL)	225±38	225±35	*0.91*	225±38	222±33	*0.51*	225±38	222±34	*0.47*	226±38
LDL-cholesterol (mg/dL)	146±35	147±32	*0.55*	146±34	147±31	*0.69*	146±34	147±31	*0.55*	147±34
HDL-cholesterol (mg/dL)	46±12	47±12	*0.73*	47±12	46±12	*0.73*	46±12	46±12	*0.73*	47±12
Systolic BP (mm Hg)	136.0±19.1	132.6±19.3	*0.04*	135.5±19.1	131.4±19.7	*0.04*	135.7±19.2	130.9±19.3	*0.04*	134.5±18.8
Diastolic BP (mm Hg)	84.2±12.3	82.9±12.9	*0.11*	84.1±12.4	82.1±12.9	*0.10*	84.3±12.4	81.9±12.7	*0.11*	83.5±12.4

Data are expressed as means ± SD or percentages; *T-test for continuous variables and Chi-square test for categorical variables. + : carriers of at least one allele. BP: bl

Suppl Table 2. Baseline characteristics of white controls according to the *PPARG* C-689T genotype in the ADVANCE Study

	Men			Women		
	CC	T+	*p**	CC	T+	*p**
N	326	97		274	86	
Age (y)	65.9 ± 3.4	65.2 ± 2.8	*0.06*	61.9 ± 6.7	60.4 ± 7.4	*0.08*
BMI (kg/m²)	28.4 ± 4.6	28.2 ± 3.7	*0.61*	27.5 ± 6.6	27.4 ± 5.7	*0.87*
Waist circumference (cm)	99.5 ± 13.1	99.4 ± 10.8	*0.92*	83.6 ± 13.9	85.0 ± 13.1	*0.41*
Activity level (%)	62.0	65.0	*0.59*	65.3	58.1	*0.23*
Current smoking (%)	6.7	7.2	*0.87*	8.4	9.3	*0.79*
Alcohol consumption (g/week)	50 (110)	60 (150)	*0.52*	20 (60)	20 (60)	*0.77*
History of hypertension (%)	51.2	51.5	*0.96*	42.1	38.6	*0.57*
History of dyslipidemia (%)	26.1	24.7	*0.79*	13.1	8.1	*0.21*
History of diabetes (%)	15.6	14.4	*0.77*	5.5	4.7	*0.77*
Total cholesterol (mg/dL)	200 ± 33	206 ± 38	*0.15*	211.0 ± 35.3	207 ± 35	*0.36*
LDL-cholesterol (mg/dL)	123 ± 29	126 ± 32	*0.36*	124.3 ± 30.0	123 ± 29	*0.78*
HDL-cholesterol (mg/dL)	49 ± 13	50 ± 15	*0.09*	62 ± 17	59 ± 14	*0.05*
Systolic BP (mm Hg)	131 ± 16	126 ± 16	*0.75*	124.9 ± 20.0	120 ± 19	*0.06*
Diastolic BP (mm Hg)	75 ± 8	75 ± 9	*0.77*	71.6 ± 9.2	71 ± 8	*0.49*

Data are expressed as means ± SD or percentages; * T-test for continuous variables and Chi-square test for categorical variables. + : carriers of at least one allele. BP: blood pressure.

Suppl Table 3. Baseline characteristics of white controls according to the *PPARG* C-681G genotype in the ADVANCE Study

	Men			Women		
	CC	G+	*p**	CC	G+	*p**
N	238	182		189	170	
Age (y)	65.9 ± 3.7	65.6 ± 2.7	*0.27*	62.0 ± 6.7	61.0 ± 7.1	*0.17*
BMI (kg/m²)	28.5 ± 4.9	28.2 ± 3.9	*0.57*	27.5 ± 7.0	27.4 ± 5.8	*0.92*
Waist circumference (cm)	99.9 ± 13.6	99.2 ± 11.6	*0.66*	83.5 ± 14.6	84.1 ± 12.8	*0.68*
Activity level (%)	60.1	66.5	*0.18*	67.7	58.8	*0.08*
Current smoking (%)	6.3	7.7	*0.58*	9	8.2	*0.80*
Alcohol consumption (g/week)	50 (110)	50 (140)	*0.69*	20 (70)	20 (60)	*0.86*
History of hypertension (%)	51.3	51.1	*0.97*	43.9	37.6	*0.23*
History of dyslipidemia (%)	25.6	26.4	*0.86*	13.8	10	*0.27*
History of diabetes (%)	15.5	15.4	*0.96*	5.8	4.7	*0.64*
Total cholesterol (mg/dL)	201 ± 34	203 ± 35	*0.43*	210 ± 37	210 ± 34	*0.91*
LDL-cholesterol (mg/dL)	123 ± 29	125 ± 30	*0.35*	123 ± 32	125 ± 28	*0.60*
HDL-cholesterol (mg/dL)	49 ± 13	49 ± 14	*0.69*	63 ± 16	61 ± 17	*0.27*
Systolic BP (mm Hg)	131 ± 15	130 ± 16	*0.86*	125 ± 20	122 ± 19	*0.09*
Diastolic BP (mm Hg)	75 ± 8	75 ± 9	*0.97*	72 ± 9	71 ± 9	*0.23*

Data are expressed as means ± SD or percentages; * T-test for continuous variables and Chi-square test for categorical variables. + : carriers of at least one allele. BP: blood pressure.

Suppl Table 4. Baseline characteristics of white controls according to the *PPARG* Pro12Ala genotype in the ADVANCE Study

	**Men**			**Women**		
	CC	G+	*p**	CC	G+	*p**
N	330	96		275	87	
Age (y)	65.9 ± 3.4	65.1 ± 2.7	*0.03*	61.9 ± 6.7	60.3 ± 7.4	*0.06*
BMI (kg/m²)	28.4 ± 4.6	28.1 ± 3.8	*0.57*	27.6 ± 6.6	27.2 ± 5.6	*0.62*
Waist circumference (cm)	99.6 ± 13.1	99.3 ± 11.0	*0.83*	83.7 ± 14.0	84.3 ± 13.0	*0.70*
Activity level (%)	61.8	65.6	*0.49*	65.5	55.2	*0.08*
Current smoking (%)	6.7	7.3	*0.83*	8.4	9.2	*0.81*
Alcohol consumption (g/week)	50 (110)	60 (150)	*0.46*	10 (60)	20 (60)	*0.58*
History of hypertension (%)	51.5	51	*0.93*	44	34.5	*0.12*
History of dyslipidemia (%)	26.4	24	*0.63*	13.1	8	*0.20*
History of diabetes (%)	16.1	13.5	*0.55*	5.5	4.6	*0.75*
Total cholesterol (mg/dL)	200 ± 33	207 ± 37	*0.09*	211 ± 35	209 ± 36	*0.66*
LDL-cholesterol (mg/dL)	123 ± 29	127 ± 31	*0.28*	124 ± 30	124 ± 30	*0.90*
HDL-cholesterol (mg/dL)	49 ± 13	50 ± 15	*0.27*	63 ± 17	60 ± 14	*0.07*
Systolic BP (mm Hg)	131 ± 16	130 ± 16	*0.79*	125 ± 20	120 ± 18	*0.05*
Diastolic BP (mm Hg)	75 ± 8	75 ± 9	*0.72*	71 ± 9	71 ± 8	*0.66*

Data are expressed as means ± SD or percentages; * T-test for continuous variables and Chi-square test for categorical variables. + : carriers of at least one allele. BP: blood pressure.

Suppl Table 5. Baseline characteristics of white controls according to the *PPARG* C1431T genotype in the ADVANCE Study

	Men			Women		
	CC	T+	*p**	CC	T+	*p**
N	325	101		278	88	
Age (y)	65.9 ± 3.5	65.2 ± 2.6	*0.07*	61.3 ± 7.0	62.0 ± 6.6	*0.38*
BMI (kg/m²)	28.4 ± 4.6	28.1 ± 3.9	*0.55*	27.5 ± 6.5	27.1 ± 5.8	*0.59*
Waist circumference (cm)	99.7 ± 13.1	99.0 ± 11.2	*0.64*	83.8 ± 13.8	83.9 ± 13.4	*0.96*
Activity level (%)	61.2	67.3	*0.27*	63.7	62.5	*0.84*
Current smoking (%)	6.8	6.9	*0.95*	7.9	10.2	*0.50*
Alcohol consumption (g/week)	50 (110)	60 (200)	*0.40*	10 (60)	20 (70)	*0.12*
History of hypertension (%)	51.1	52.5	*0.81*	42.1	38.6	*0.57*
History of dyslipidemia (%)	26.2	24.8	*0.78*	12.2	10.2	*0.61*
History of diabetes (%)	15.1	16.8	*0.67*	4.7	6.8	*0.43*
Total cholesterol (mg/dL)	202 ± 33	202 ± 38	*0.88*	210 ± 35	211 ± 35	*0.89*
LDL-cholesterol (mg/dL)	124 ± 29	122.4 ± 30.7	*0.62*	124 ± 31	126 ± 27	*0.58*
HDL-cholesterol (mg/dL)	49 ± 13	50.1 ± 14.7	*0.39*	62 ± 16	60 ± 17	*0.35*
Systolic BP (mm Hg)	131 ± 16	130.0 ± 16.2	*0.67*	124 ± 20	123 ± 19	*0.67*
Diastolic BP (mm Hg)	75 ± 8	75.5 ± 9.0	*0.70*	72 ± 9	70 ± 9	*0.20*

Data are expressed as means ± SD or percentages; * T-test for continuous variables and Chi-square test for categorical variables. + : carriers of at least one allele. BP: blood pressure.
